# A Process Evaluation of a Web-Based Mental Health Portal (WalkAlong) Using Google Analytics

**DOI:** 10.2196/mental.8594

**Published:** 2018-08-20

**Authors:** Michael Jae Song, John Ward, Fiona Choi, Mohammadali Nikoo, Anastasia Frank, Farhud Shams, Katarina Tabi, Daniel Vigo, Michael Krausz

**Affiliations:** ^1^ Department of Psychiatry University of British Columbia Vancouver, BC Canada; ^2^ Centre for Health Evaluation and Outcome Sciences St Paul's Hospital Vancouver, BC Canada; ^3^ Department of Pharmacology Faculty of Medicine Masaryk University Brno Czech Republic; ^4^ Centre for Applied Research in Mental Health and Addictions Faculty of Health Sciences Simon Fraser University Burnaby, BC Canada; ^5^ School of Population and Public Health University of British Columbia Vancouver, BC Canada

**Keywords:** evaluation, Google Analytics, mental health, website

## Abstract

**Background:**

Despite the increasing amount of research on Web-based mental health interventions with proven efficacy, high attrition rates decrease their effectiveness. Continued process evaluations should be performed to maximize the target population’s engagement. Google Analytics has been used to evaluate various health-related Web-based programs and may also be useful for Web-based mental health programs.

**Objective:**

The objective of our study was to evaluate WalkAlong.ca, a youth-oriented mental health web-portal, using Google Analytics to inform the improvement strategy for the platform and to demonstrate the use of Google Analytics as a tool for process evaluation of Web-based mental health interventions.

**Methods:**

Google Analytics was used to monitor user activity during WalkAlong’s first year of operation (Nov 13, 2013-Nov 13, 2014). Selected Google Analytic variables were overall website engagement including pages visited per session, utilization rate of specific features, and user access mode and location.

**Results:**

The results included data from 3076 users viewing 29,299 pages. Users spent less average time on Mindsteps (0 minute 35 seconds) and self-exercises (1 minute 08 seconds), which are important self-help tools, compared with that on the Screener tool (3 minutes 4 seconds). Of all visitors, 82.3% (4378/5318) were desktop users, followed by 12.7 % (677/5318) mobile phone and 5.0% (263/5318) tablet users. Both direct traffic (access via URL) and referrals by email had more than 7 pages viewed per session and longer than average time of 6 minutes per session. The majority of users (67%) accessed the platform from Canada.

**Conclusions:**

Engagement and feature utilization rates are higher among people who receive personal invitations to visit the site. Low utilization rates with specific features offer a starting place for further exploration of users in order to identify the root cause. The data provided by Google Analytics, although informative, can be supplemented by other evaluation methods (ie, qualitative methods) in order to better determine the modifications required to improve user engagement. Google Analytics can play a vital role in highlighting the preferences of those using Web-based mental health tools.

## Introduction

As technologies such as internet, mobile phones, and computers have become ubiquitous, Web-based interventions have become one of the major treatment and preventative tools for mental disorders. More than 100 randomized controlled trials have been published to demonstrate the efficacy of internet interventions for psychiatric disorders [1]. These tools have been shown to be effective for a range of mental illnesses including panic disorder, depression, posttraumatic stress disorder (PTSD), perceived stress in schizophrenia, stress, insomnia, and eating disorders [2]. The potential of Web-based mental health interventions to effectively and efficiently treat and prevent mental illnesses has attracted many health care providers and researchers to explore using them as one of the major components of the mental health care system.

Despite their promising benefits, online mental health interventions face problems in engagement [3-5]. In relation to taking a drug, Web-based interventions are more vulnerable to disengagement as they lack close supervision, are easy to discontinue, and have no immediate health benefits [5]. Dropout rates for Web-based interventions are up to 50% in guided interventions and up to 74% in unguided interventions [6,7]. With such high dropout rates, these interventions may provide only limited outcomes regardless of their proven efficacy [8,9].

Engagement is especially challenging in the context of mental health. Dropout from traditional treatment among those with mental illness is already a cause for concern [10]. The chronic nature of mental illness requires frequent assessments in conjunction with long-term management that is tailored to individuals’ needs [11]. In addition, disorder-specific features such as symptom severity, emotional distress, and medication side effects have been shown to predict adherence [12,13]. In a technological context, being engaged can be described as “a category of user experience characterized by attributes of challenge, positive affect, endurability, aesthetic and sensory appeal, attention, feedback, variety or novelty, interactivity, and perceived user control” [14]. In other words, scientists and developers have the responsibility of ensuring not only the efficacy of their intervention but also user engagement. Strategies to increase user engagement may include continual platform improvement and improving outreach or marketing strategies.

A key step to improve engagement is identifying ways to improve intervention uptake in real-world settings through process evaluation [5]. A process evaluation is a type of assessment that determines whether program activities have been implemented as intended [15]. The goal is to inform strategies toward achieving optimal engagement and effectiveness of an intervention [16-18]. By understanding how users engage with the intervention, such as the specific pages visited or how long they used the website, process evaluation can inform the adaptation of the intervention in order to maximize user exposure to the tools and the knowledge available in Web-based interventions [18]. Ideally, this would involve a mixed-methods approach where both quantitative and qualitative indicators can collectively measure user perceptions or behavior [17]. However, this is not always possible for asynchronous, open-access, Web-based interventions and with limited resources.

**Figure 1:**
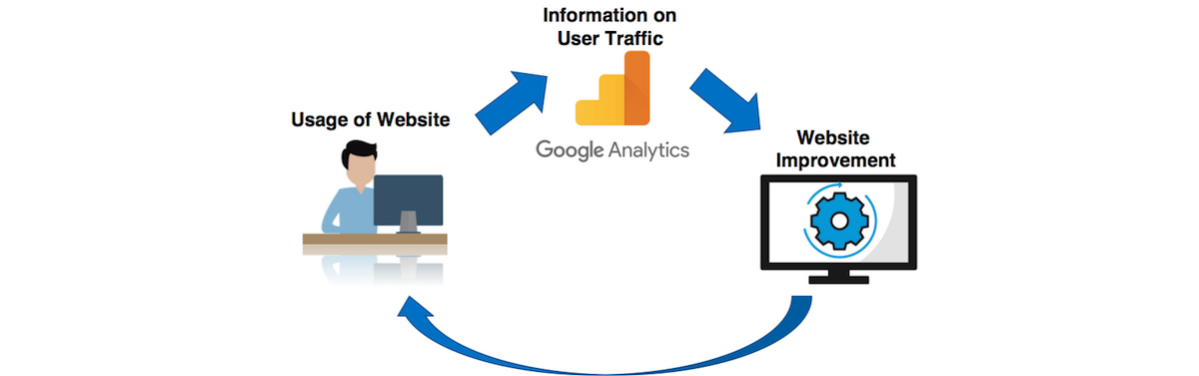
Google Analytics can be used as a tool for process evaluation by receiving information on user traffic and subsequently informing website improvement. This process can also continue as a cycle for continual improvement of the website.

In this context, one tool that can be used is Google Analytics, which is an open tool that provides free quantitative data on website usage that can be leveraged for continual website improvements (Figure 1). Although this tool is designed to provide insights from a marketing perspective, numerous variables about the webpage traffic are collected that can inform the process evaluation of Web-based interventions. Indeed, Google Analytics has already been used in health research as part of process evaluation [19-21]. For example, this tool has been used to assess the usage of a website about sexual health [19], an internet-delivered genetics education resource developed for nurses [21], and a Web-based tool to encourage the proper use of antibiotics [16], as well as websites related to osteoporosis and fractures [22], smoking cessation [23], and knowledge translation [24]. These studies have presented various indicators available from Google Analytics to show overall user engagement with their platforms. However, to our knowledge, the use of Google Analytics has not yet been demonstrated for Web-based mental health platforms that provide direct support for youth with mental health challenges. Since Web-based mental health platforms may face challenges with adherence, Google Analytics could be used to better understand user behavior as part of process evaluation and to come up with strategies that would improve adherence.

**Figure 2:**
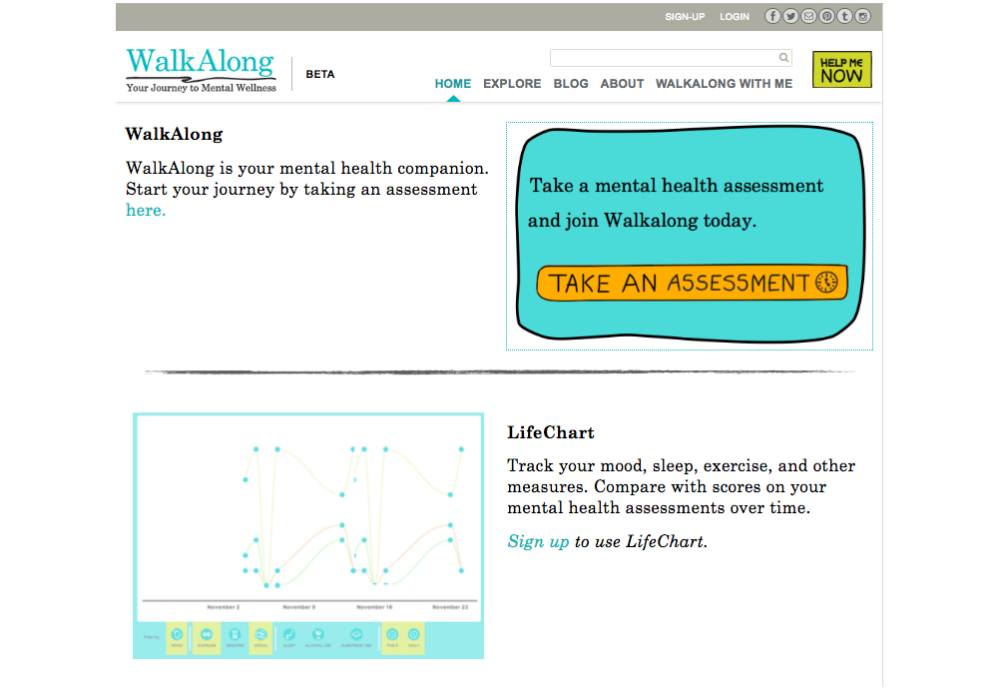
WalkAlong home page.

The goal of this study was to evaluate WalkAlong, a Web-based mental health platform, using Google Analytics. This platform is a youth-oriented mental health Web-portal designed to provide young people with tools and resources required to manage their own mental health (Figure 2; Multimedia Appendix 1). The Web-portal focuses on supporting mood and anxiety disorders and has received funding under Bell Canada’s Let’s Talk mental health initiative. The portal is freely accessible to all users with an internet connection and contains information, links to resources, and self-help tools including a description and link to MoodGYM, a Web-based resource for depression and anxiety. Self-help tools available directly on the platform include the “Mind Steps” page, which consists of regularly posted short tips for helping users get through the day, and “The Self-Help Exercises” section, displaying a menu with the individual self-help exercise pages. Assessment screeners for depression and anxiety (Patient Health Questionnaire-9 and Generalized Anxiety Disorder 7-item scale) are also available. Users may also create a password-protected, secure account that grants them access to additional resources including the PTSD CheckList—Civilian Version, community resources, and a Life-Chart tracking tool that tracks mood and behaviors.

Our objective was to use Google Analytics as a tool for conducting a process evaluation of the WalkAlong platform. As part of the process evaluation, the following evaluation questions using Google Analytics were asked: (1) How engaged (ie, time spent on the website) are users with the WalkAlong platform? (2) How can the WalkAlong platform be improved to better engage the users? (3) How can the marketing strategy be shaped to engage and reach out to more users? Another objective of this project is to extend the work to a mental health platform from other health interventions and inform website design and marketing strategies to effectively impact user behavior [19,21].

## Methods

### Google Analytics

Google Analytics was used to access user data over the first year of WalkAlong (Nov 13, 2013-Nov 13, 2014). Focusing on the first year of operation was considered the most appropriate approach in order to capture a snapshot of web traffic following the initial launch. The Google Analytics data do not contain any personally identifiable information and are presented in the form of aggregate data, making it an accessible tool used in research settings without ethical concerns [20,21].

The research team installed Google Analytics by adding a tracking tag for WalkAlong [20]. These tracking tags are snippets of JavaScript code, a computer programming language used to build websites. This code allows collection of various forms of data related to user behavior as soon as the user visits the website. The data can emanate from various avenues such as the URL of the page the user is viewing, the language or the name of the browser, and the device used to access the site. The code also collects information on the nature of the visit such as the contents viewed, length of the session, and channels used to access the platform (eg, Google, direct URL search, social media, and email link). Such information is summarized in a real-time, interactive dashboard format, which can be accessed by logging in.

### Overall Engagement

Several indicators from Google Analytics that would allow inference of a level of engagement were calculated. Such indicators include the number of returning users (n), bounce rate (%), number of pages accessed per session (n), mean session duration (minutes, seconds), and goal conversion rate (%).

The number of returning users refers to the number of sessions visited through the same client id. A high number of returning users has been used as an indicator for a strong level of engagement with the platform [21,25].

The bounce rate is the percentage of only a single page visit during a session. A high bounce rate could indicate minimal exposure to the intervention due to minimal interaction, but it could also indicate users exiting as they have found what they were looking for right away. However, generally, a low bounce rate can be considered indicative of a high overall engagement, especially for a multicomponent platform like WalkAlong [19]. For instance, there are not much available resources that would provide mental health support in the home page of the platform alone as it simply offers an overview. Users will often need to interact with various tools and webpages in order to obtain the necessary information.

The number of pages per session refers to the number of webpages within the platform that the user viewed in a single session, and the mean session duration (minutes, seconds) refers to the mean duration of time the users spent on the platform. There are limitations to ascertaining engagement through these indicators since they allow for multiple interpretations: a high number of pages per session could result from an increased engagement, but it could also result from a superficial exploration of several pages; similarly, a long session duration can result from increased engagement, but it could also result from a user keeping the webpage open while engaging in other irrelevant activities. Nevertheless, despite these caveats, traffic information provides an approximation of the level of exposure the users had with the platform [25].

The goal conversion rate measures the proportion of sessions that achieved a goal out of the total sessions. The goal was predefined as creating an account but can be defined as any activity the web developer or owner chooses (eg, buying a product). As discussed above, users who create an account are able to access more resources than those who do not (anonymous users). Thus, creating an account was assumed to indicate a stronger level of engagement. Overall, a high number of returning users, low bounce rate, high number of pages viewed per session, high mean session duration, and high goal conversion rates collectively translate to an estimate of a strong level of engagement [20,25].

### Platform Improvement

Several indicators from Google Analytics that can inform the improvement of the platform were also selected. Indicators of user behavior such as page views, mean duration of visit, and bounce rate when accessing self-help tools (eg, Mindsteps page, Self-Help Exercises page, and Screener) were analyzed. In addition, the most visited pages were observed in terms of their overall entrance rate, exit rate, and bounce rate to understand which tools or pages were most used or viewed. The entrance rate represents a proportion of sessions starting from a given page, while the exit rate represents a proportion of sessions ending from a given page. The information regarding the entrance rate may provide an understanding about which webpage is serving as the first impression for the users, and the exit rate may indicate the point when users felt disengaged or, on the contrary, had adequate information needed for the session.

Google Analytics also provided data on the type of devices used for access. Such information can allow us to consider whether developing a mobile app for WalkAlong would be helpful or not. The three main devices of interest to the current investigation were desktops, tablets, and mobile phones (counted here as mobile devices).

### Marketing Strategy

Google Analytics was also used to inform our marketing strategy, with the goal of reaching as many users as possible. At the outset, the research team had reached out to different youth and university organizations, especially around Vancouver. Twitter and a Facebook accounts were also created to spread awareness about the platform. To improve the marketing strategy, the channels used to access the platform were observed. The channels are direct link (ie, typing the web URL directly into a browser); organic search (ie, entry through a search engine); and referrals via another website, via social media, and via email. Understanding which channels are underutilized and which channel results in the highest level of engagement can help improve the marketing strategy. Locations of users from different countries around the world were also observed.

## Results

### Overall Engagement

**Figure 3:**
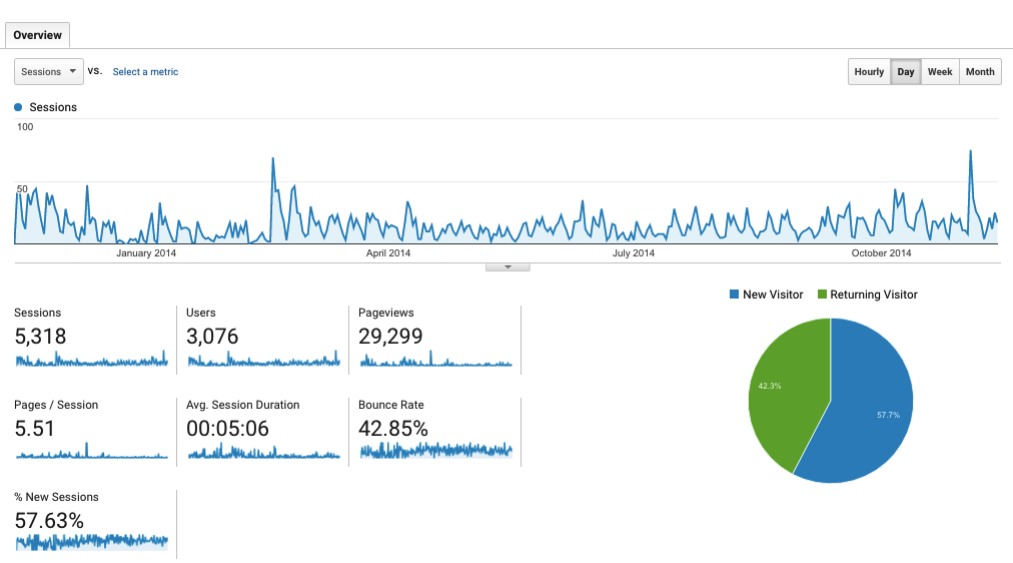
WalkAlong overview presented in Google Analytics.

The first year of operation for the WalkAlong platform saw a total of 3076 users, amounting to 5318 sessions and 29,299 page views (Figure 3). On average, users visited 5.51 pages per session with an average session duration of 5 minutes 6 seconds. The average bounce rate was 42.9% where users only viewed a single page; 31.7% (976/3076) users created an account (goal completion).

In terms of the frequency of visits, 80% (4259/5318) of sessions came from users visiting less than nine times, indicating a level of disengagement after a certain number of visits (Table 1).

The number of sessions during the study period decreased with increasing number of visits. However, there was a slight increase at the upper end of sessions from high-frequency visits: 5.8% (311/5318) accounted for 26-50 visits and 4.7% (250/5318) accounted for 51-100 visits over the time period. The number of sessions also decreased with longer session durations (Table 2). These results indicate that the majority of sessions or 65.4% (3477/531) resulted in disengagement within the first minute.

**Table 1:** Proportion of total sessions and number of visits.

Visits	Sessions (N=5318), n (%)
1	3066 (57.65)
2	550 (10.3)
3	241 (4.5)
4	139 (2.6)
5	100 (1.9)
6	67 (1.3)
7	50 (0.9)
8	46 (0.9)
9-14	180 (3.4)
15-25	205 (3.9)
26-50	311 (5.8)
51-100	250 (4.7)
101-200	112 (2.1)
201+	1 (0.0)

**Table 2:** Duration of session.

Session duration (in minutes)	Sessions (N=5318), n (%)
≤1	3477 (65.4%)
1-3	527 (9.9%)
3-10	581 (10.9%)
>10	733 (13.8%)

### Platform Improvement

Visits to the Mindsteps tool comprised 11.9% (3493/29,299) of total page views, with mean duration spent of 35 seconds. Visits to the Self-Help Exercises page comprised 6.13% (1797/29,299), with mean duration spent of 1 minute 8 seconds. Visits to the Screener comprised only 3.36% (983/29,299) of the total page views, but the mean duration spent on the Screener was 3 minutes 4 seconds. Table 3 presents the entrance and exit rates for the most viewed pages, which included the Self-Help Exercises, Mindsteps, and the Screener. The WalkAlong home page, which acts as the landing page, accounted for 65.6% (3487/5308) of all entries.

A list of devices used by WalkAlong’s users to access the site is presented in Table 4, indicating that the platform was accessed mostly via desktops (4378/5318, 82.3%). Furthermore, sessions completed via desktops had a lower bounce rate (39.6%), higher pages per session (6.17), and a higher conversion rate (22.7%) than those completed via other devices.

### Marketing Strategy

Direct traffic accounted for the highest proportion (2420/5318, 45.5%) of all visits to the site (Table 5). The combination of high bounce rate (50.4%) and low conversion rate (11.3%) among organic searches suggests that these particular users did not engage much with the site content. Visits via referrals or social media sites had relatively less traffic at 16.0% (849/5318) and 13.5% (717/5318), respectively, and both had more than 45% bounce rates. Although emails accounted for promoting only 1.4% of all sessions, they had a low bounce rate of 17% and a long average session duration of 5 minutes 6 seconds with a conversion rate of 25%, collectively indicating a relatively strong engagement.

**Figure 4:**
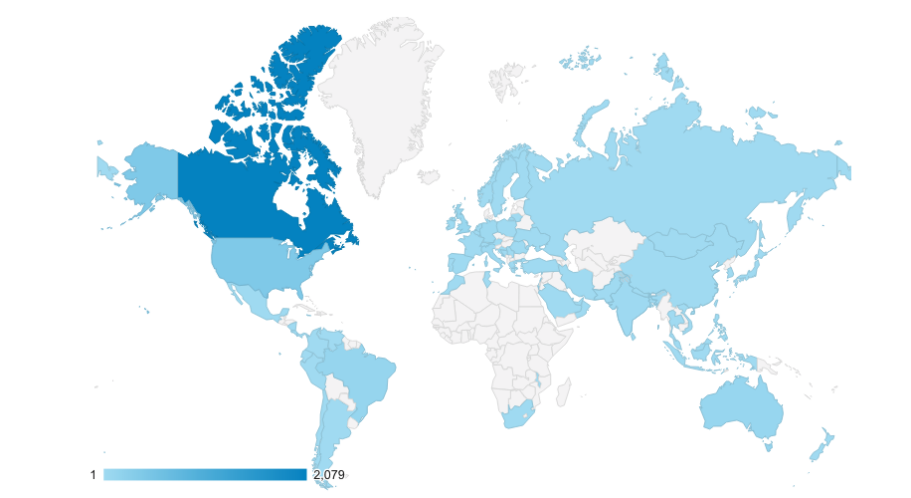
Map overlay about locations of users from Google Analytics.

Approximately two-thirds or 67.6% (2079/3076) of the users belonged to Canada. Users from Canada also had a relatively low bounce rate (34.35%), high number of pages viewed per session (6.57 pages per session), and long session duration (6:10). However, the users accessed the platform from around the world (Figure 4).

**Table 3:** Entrance and exit rates for the most viewed pages.

Page	Entrances n (%)^a^	Exits (%)^b^	Bounce rate (%)
Home page	3487 (65.6)	29.2	37.6
Depression in Canada	115 (2.2)	73.4	86.1
Self-Help Exercises	70 (1.3)	14	47.9
Mindsteps	62 (1.2)	5.4	24.2
Screener	55 (1.0)	32.0	50.9

^a^The numbers do not add up to 100% because only several of the most viewed pages are included in the table.

^b^The exit rate is calculated by the number of exits/number of times that page was viewed. Thus, the added percentages are higher than 100% where each row has different number of exits and the number of pages viewed.

**Table 4:** Devices used to access WalkAlong.

Device	Sessions (N=5318), n (%)	Bounce rate (%)	Pages per session, n	Mean session duration	Conversion rate (%)
Desktop	4378 (82.32)	39.6	6.17	5 min 43 s	22.7
Mobile phone	677 (12.7)	61.6	2.15	1 min 53 s	7.2
Tablet	263 (5.0)	48.7	3.08	3 min 15 s	11.8

**Table 5:** Proportion of total sessions for each type of channel.

Channels	Sessions (N=5318), n (%)	Bounce rate (%)	Pages per session, n	Mean session duration	Conversion rate (%)
Direct Traffic	2,420 (45.51)	36.6	7.4	6 min 38 s	24.4
Organic Search	1,256 (23.62)	50.4	3.5	3 min 42 s	11.3
Referrals	849 (16.0)	46.9	3.5	3 min 46 s	22.6
Social Media	717 (13.5)	48.8	4.6	3 min 44 s	18.0
Email	76 (1)	17.1	10.5	7 min 39 s	25.0

## Discussion

### Overall Engagement

The first year of operation for the WalkAlong platform saw a total of 3076 users, amounting to 5318 sessions of 5 minutes 6 seconds on average, 29,299 page views, and 31.7% goal completion rate. However, the high proportion of sessions comprising first visits and short session durations suggest a degree of disengagement for the users (Tables 1 and 2). Although the reason for the disengagement is unclear, as it could also be due to acquiring information that is needed early on, this finding does not contradict the pre-existing concerns about lack of engagement evidenced by Web-based mental health interventions [3-5]. These results call for further efforts to continuously improve the platform to be more engaging.

### Platform Improvement

The platform can also be improved based on user behavior. For instance, there could be further efforts to improve engagement with tools such as the “Mindsteps” and “Self-Help Exercises.” The mean duration of 35 seconds spent on Mindsteps or 1 minute 8 seconds spent on Self-Help Exercises may be deemed too short considering that they are important components of WalkAlong intended to improve mental health outcomes. The relatively short time spent on “Mindsteps” and “Self-Help Exercises” needs to be addressed using better engagement strategies such as improved web design or involving youth to be part of the design process [26]. The WalkAlong home page had the highest entrance rate, indicating that users started their session from this page. This landing information reinforces the role of home page serving as the first impression, and determining the subsequent user behavior [27].

In terms of the devices used to access the WalkAlong platform, the site was viewed mostly via desktops. However, as the usage of mobile phones is ubiquitous among youth, future improvements in WalkAlong may benefit from making the platform more accessible and engaging for mobile phone users [28,29]. A next step could involve developing a native mobile phone app version of the WalkAlong website. This could allow users to access the platform wherever they are without the need of a desktop.

### Marketing Strategy

The data indicate that some form of personal referrals indicated by either email or prior knowledge of the URL (direct traffic) results in a relatively stronger engagement (ie, longer average duration, more pages viewed per session, etc) than less personal channels such as referrals through social media or organic searches. In other words, direct referrals such as word-of-mouth strategies among peers could help increase the number of engaged users [30]. This may also include engaging with health care professionals so they can share the platform’s URL with their clients.

When observing the location of the users, 67% (2079/3076) users belonged to Canada. This finding aligns with the limited marketing strategy used in Vancouver. WalkAlong can be used by all English-speaking countries, but it can also be used as a template for other platform developments internationally. The WalkAlong team could consider spreading awareness beyond Canada to ensure that such a resource is available to as much youth population as possible.

### Using Google Analytics as a Tool for Process Evaluation

Although Google Analytics has provided promising data on the usage patterns of the WalkAlong platform, the tool should be used with careful consideration. For example, comparing the results across various interventions is currently difficult as they serve different purposes with different standards in the number of users, sessions, and page views [19,25]. For instance, Crutzen et al’s website about sexual health showed 850,895 visitors with 5 minutes 6 seconds of average visiting time from March 2009 to December 2010 [19]. It can be assumed that this is a much higher number of visitors with similar duration of visiting time. However, with different periods of time being evaluated for a website serving different purposes, it is difficult to establish the standards for success. Instead, the overall engagement numbers, in particular, may be used to observe trends in usage across different time periods where continual evaluation of the platform is encouraged.

Google Analytics also conforms to a marketing perspective of Web-based behavior rather than to a full evaluation of user behavior [20]. Thus, some variables and information available may not reflect scientific inquiry. This is further complicated by the fact that Google Analytics provides aggregate data where testing of statistical significance for rigorous research purposes can be difficult. Furthermore, the validity of various indicators in measuring user engagement is yet to be established. The number of users may be inaccurate as a new client id is given every time the user deletes the browser cookies, switches devices, or uses a different browser. This may result in the same user being counted as a new user [20]. In addition, long session durations may not, in fact, indicate that users are engaging with the content or a high bounce rate may not indicate that users exit the page due to disinterest quickly, as they could have just quickly found the relevant information they needed. A more detailed analysis of longitudinal user data or a mixed-method assessment to supplement Google Analytics will be important for a more comprehensive process evaluation [31]. For instance, as this study looks only at the first year of operation of WalkAlong, a future study could examine subsequent years comparing internet traffic following changes to the platform, some of which are based on the recommendations mentioned in this paper. Overall, Google Analytics, in combination with other evaluation methods (eg, focus groups, surveys, etc), will provide more accurate interpretations when conducting process evaluation.

### Conclusion

Google Analytics was helpful in informing the process evaluation of an open-access Web-based mental health platform. The process evaluation provided information about marketing strategies as well as the aspects of the platform that required improvement. Ideas for future improvements may include marketing the WalkAlong platform outside Canada to get more users from other countries and making the platform more accessible and engaging for mobile users. The rich aggregate data, when combined with other evaluation methods, may provide more accurate interpretations to reinforce or challenge these ideas. Therefore, future studies should focus on developing a mixed methodology that includes Google Analytics to conduct process evaluation of open-access Web-based mental health platforms. With high-quality process evaluation, Web-based mental health interventions may be not only effective but also engaging.

## Appendix

Multimedia Appendix 1 Screenshots of pages from WalkAlong.

## Abbreviations

PTSD: posttraumatic stress disorder
