# Supplementary figures and images for: A Process Evaluation of a Web-Based Mental Health Portal (WalkAlong) Using Google Analytics
Source: JMIR Ment Health. 2018 Aug 20;5(3):e50. doi: 10.2196/mental.8594 (PMC6121139; doi:10.2196/mental.8594)

## Slide 1
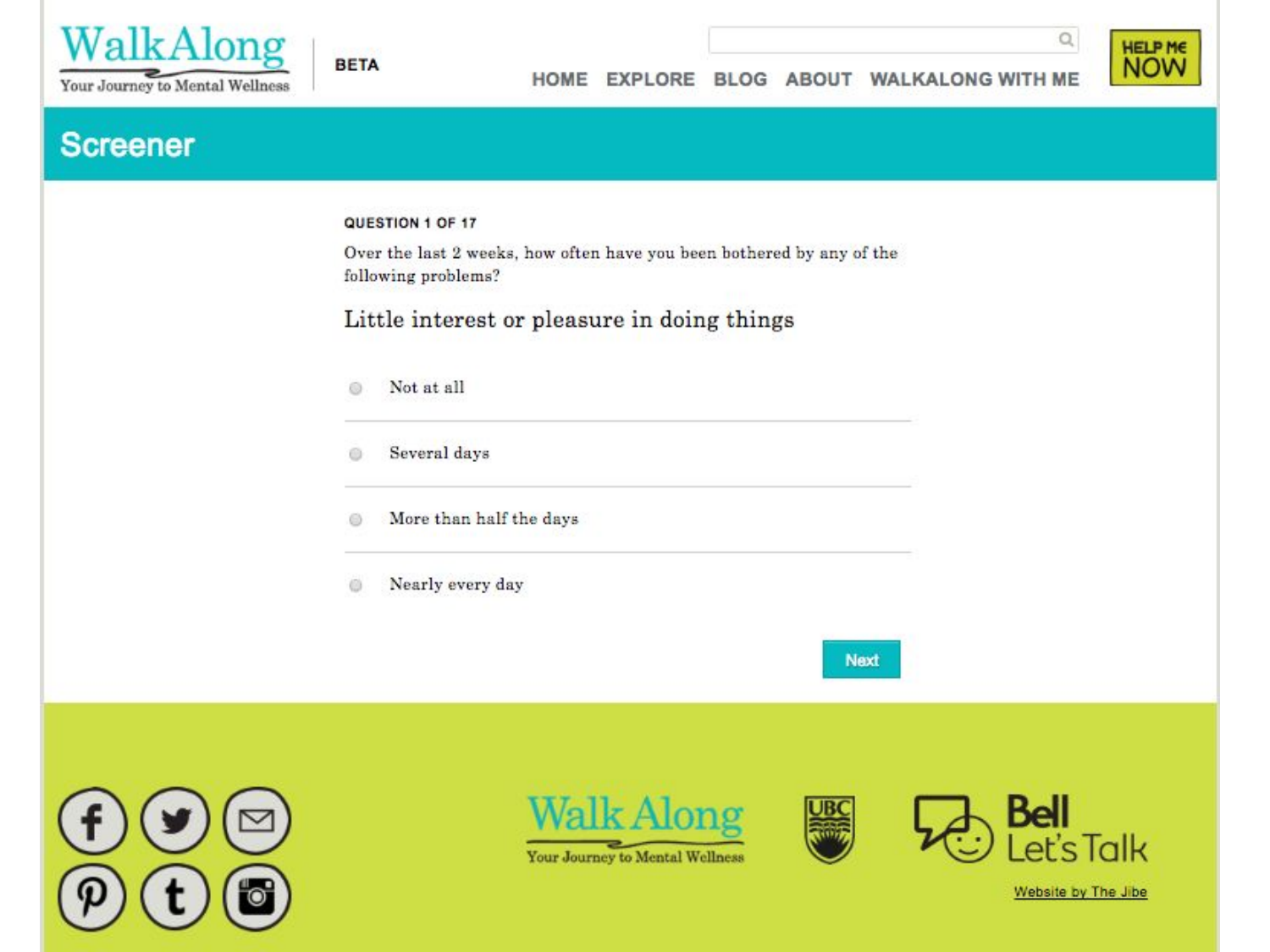

## Slide 2
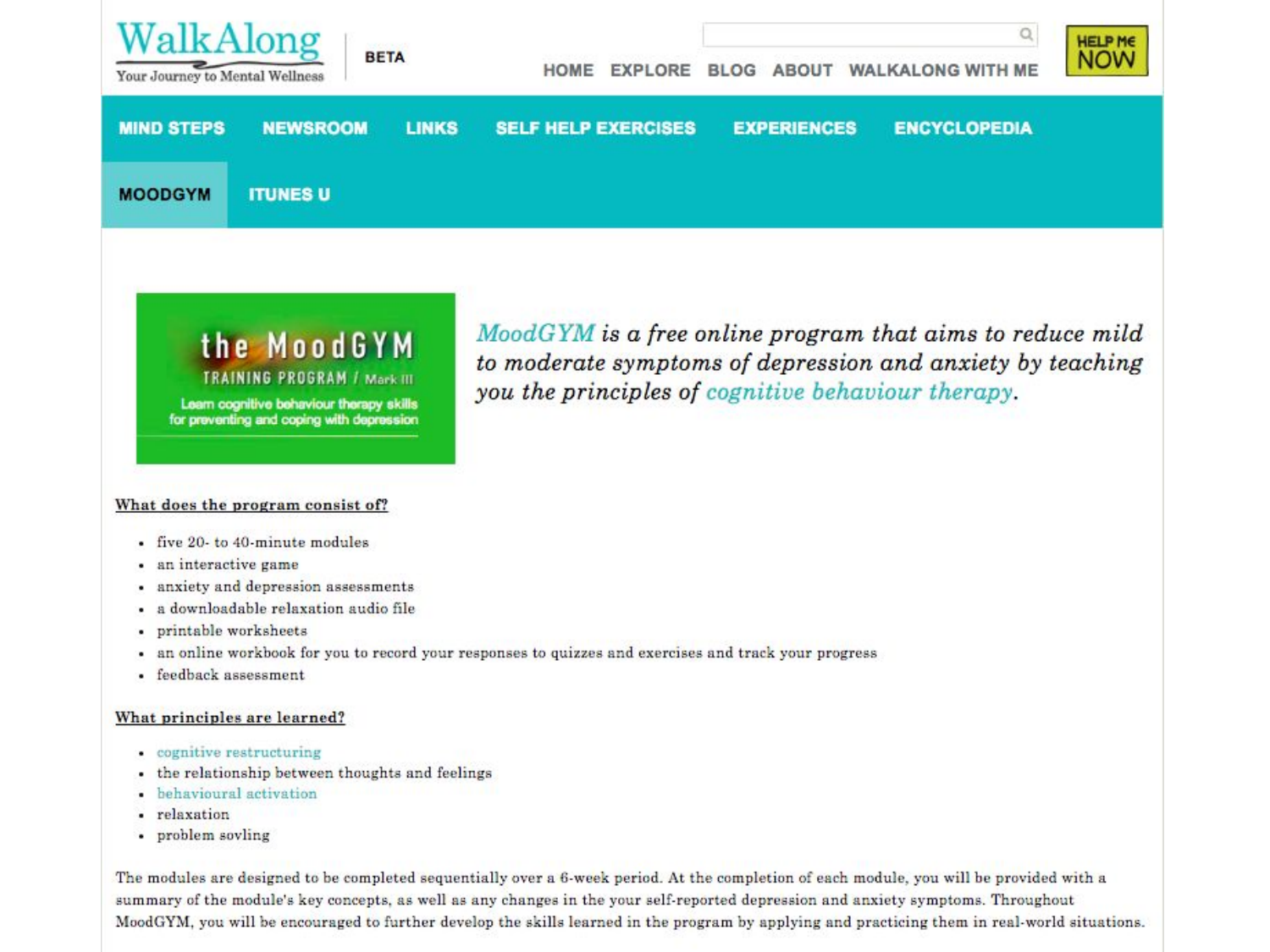

## Slide 3
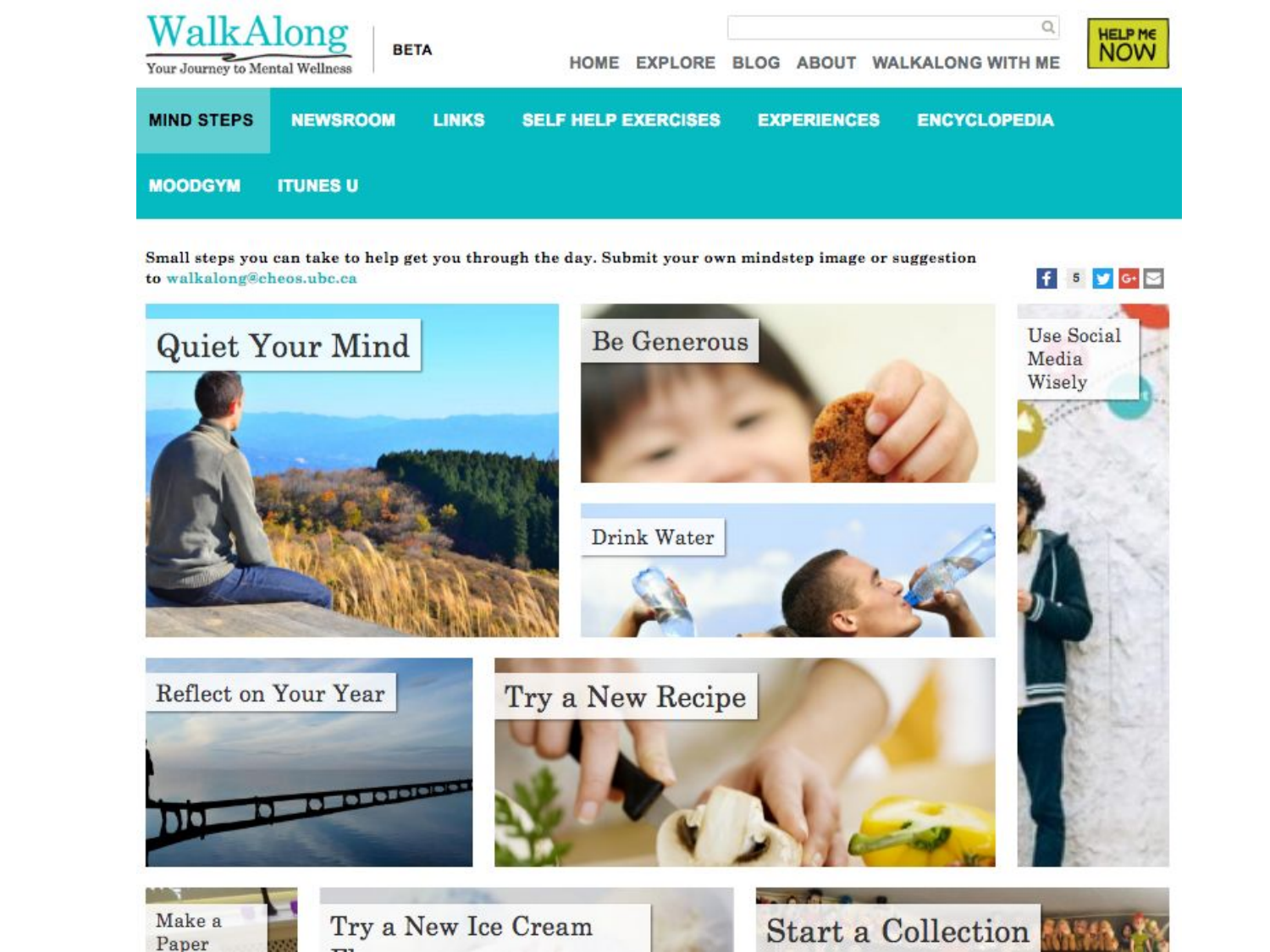

## Slide 4
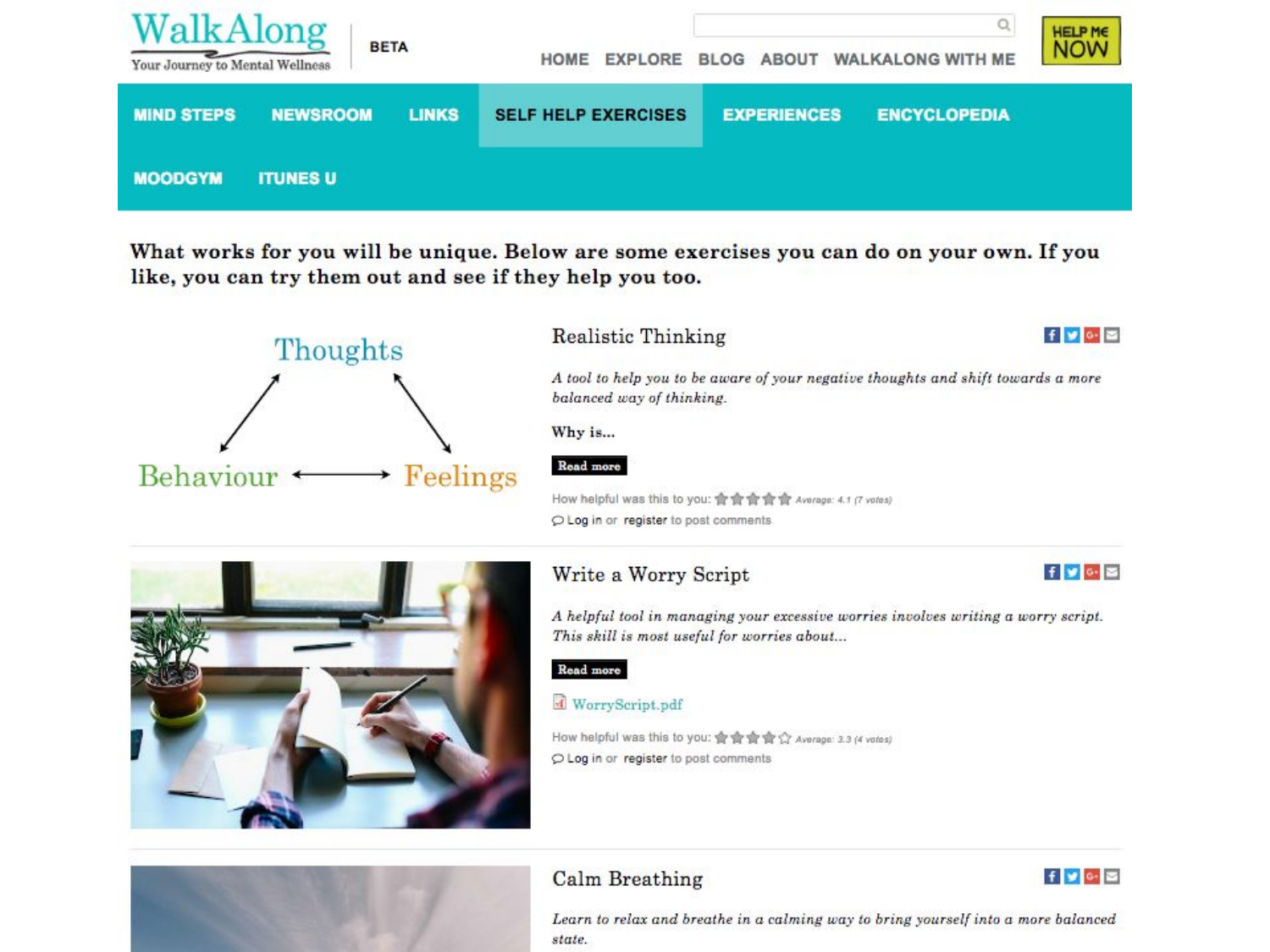

## Slide 5
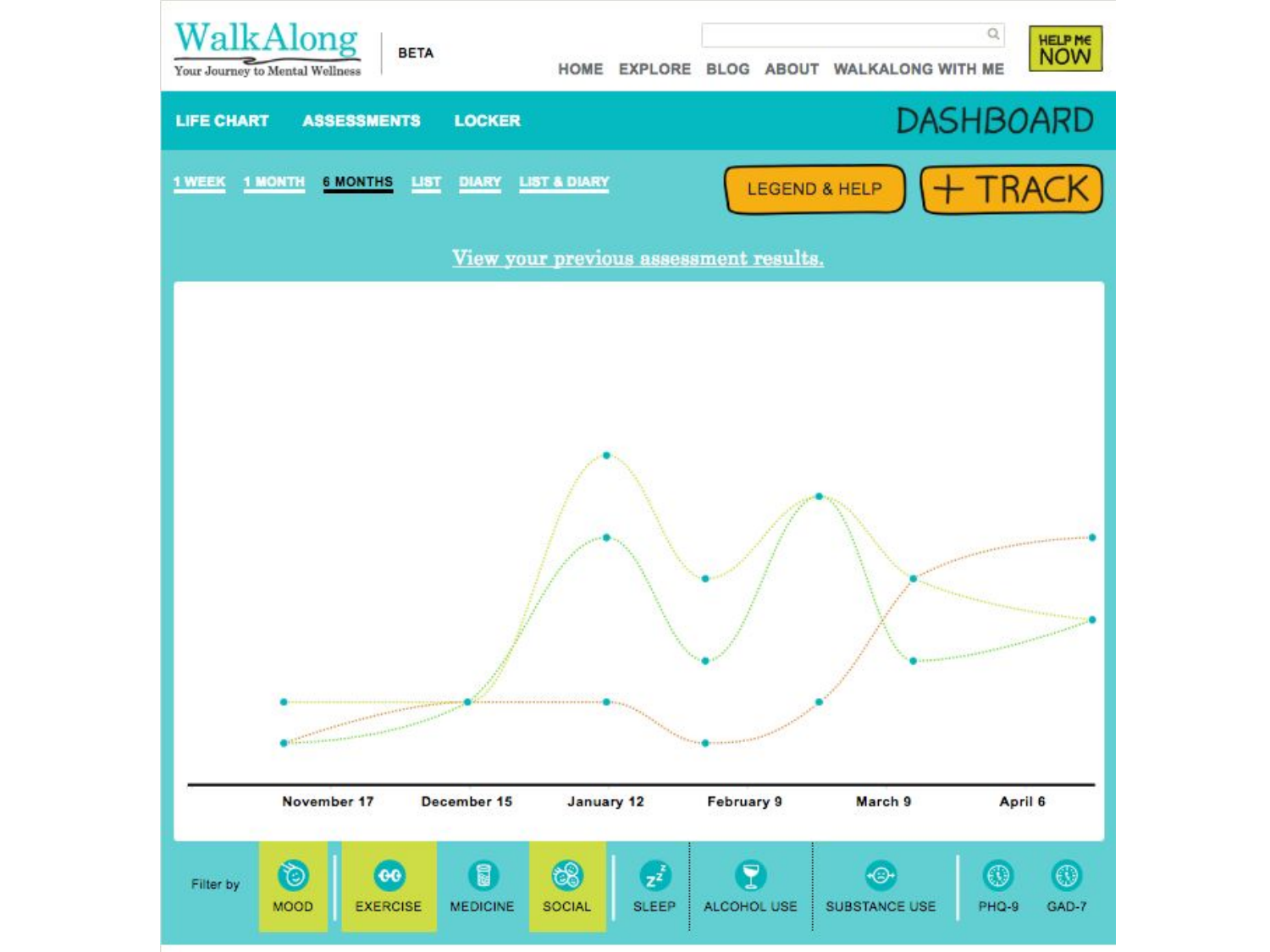

Supplement: Multimedia Appendix 1 [file mental_v5i3e50_app1.pptx]
